# Primary desmoplastic small round cell tumor of the submandibular gland: a case report and literature review

**DOI:** 10.1186/s13000-021-01183-3

**Published:** 2022-01-07

**Authors:** Jiayu Zhou, Qingling Li, Baihua Luo, Xiaodan Fu, Chunlin Ou, Xiaomei Gao, Zhijie Xu, Deyun Feng, Keda Yang

**Affiliations:** Department of Pathology, Xiangya Hospital, Central South University, Changsha, China

**Keywords:** Desmoplastic small round cell tumor, Submandibular gland, Small round blue cell tumors, Fine needle aspiration cytology, Literature review

## Abstract

**Background:**

Desmoplastic small round cell tumor (DSRCT) is a sporadic, highly malignant tumor with a poor prognosis. The abdomen and pelvis have been reported as the primary localization sites. However, to the best of our knowledge, there are few reports on primary DSRCT in the submandibular gland.

**Case presentation:**

We report a case of a 26-year-old Chinese man with a mass in the right submandibular gland. Imaging studies showed a hypoechoic mass in the right submandibular region. Intraoperative pathology revealed that the tumor tissue was composed of small round tumor cells and a dense desmoplastic stroma. On immunostaining, the tumor cells showed markers of epithelial, mesenchymal, myogenic, and neural differentiation. The *EWSR1* gene rearrangement was detected by fluorescence in situ hybridization. Based on the overall morphological features and immunohistochemical findings, a final diagnosis of DSRCT was made. The patient was treated with comprehensive anti-tumor therapy mainly based on radiotherapy and chemotherapy.

**Conclusions:**

DSRCT is an uncommon malignant neoplasm with rare submandibular gland involvement. In this report, we have described a case of DSRCT in the submandibular gland and reviewed the literature on DSRCT over the past 5 years. Considering the importance of differential diagnosis between DSRCT, especially with rare extra-peritoneal involvement, and small round blue cell tumors, a full recognition of the clinicopathological features will help to better diagnose this neoplasm.

**Supplementary Information:**

The online version contains supplementary material available at 10.1186/s13000-021-01183-3.

## Background

Desmoplastic small round cell tumor (DSRCT) is an extremely rare and aggressive neoplasm that most commonly affects adolescents and young adults with a male predominance [1–7]. DSRCT preferentially involves the abdominal and pelvic cavities [8–11]. DSRCT in the pleura, lung, eye, ear, and testis has been reported only in a few cases (< 5%) [3–7], but it is not specifically associated with any organ. Clinical symptoms are usually associated with the tumor site and lack specificity. DSRCT can metastasize in the early stage and quickly recurs, despite treatment [12–15]. Hence, its prognosis is poor. Herein, we have presented a rare case of primary DSRCT in the submandibular gland. We have described and summarized the clinicopathological and cytological features of DSRCT, in addition to discussing its diagnosis and treatment based on the literature review.

## Case presentation

### Clinical history

**Fig. 1:**
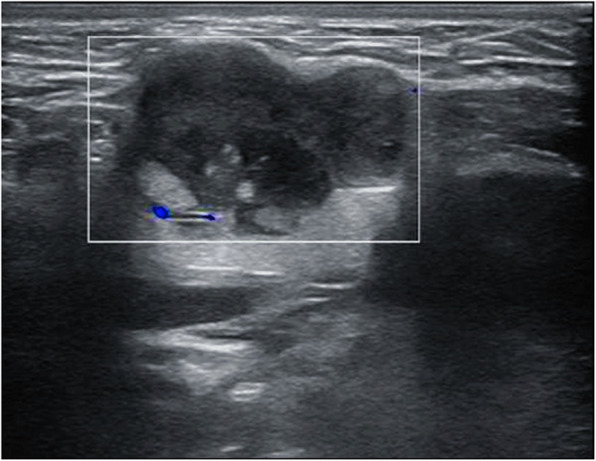
Cervical US: A hypoechoic, well-defined mass measuring 28 mm × 18 mm located in the right submandibular region; no obvious blood flow signal was observed in the lesion

A 26-year-old Chinese man with a chief complaint of a mass in the right submandibular region for the past 1 year was admitted to Xiangya Hospital, Central South University, Hunan, China. He had no significant past medical or family history. Routine physical and laboratory examinations were performed. Ultrasonography revealed a hypoechoic mass measuring approximately 28 mm × 18 mm in the right submandibular region, with an irregular shape and clear boundary (Fig. 1). Abdominal computed tomography (CT) scan revealed no other lesion. There was no evidence of metastasis to the local or distant organs. Hence, lumpectomy was performed under general anesthesia.

### Pathology

**Fig. 2:**
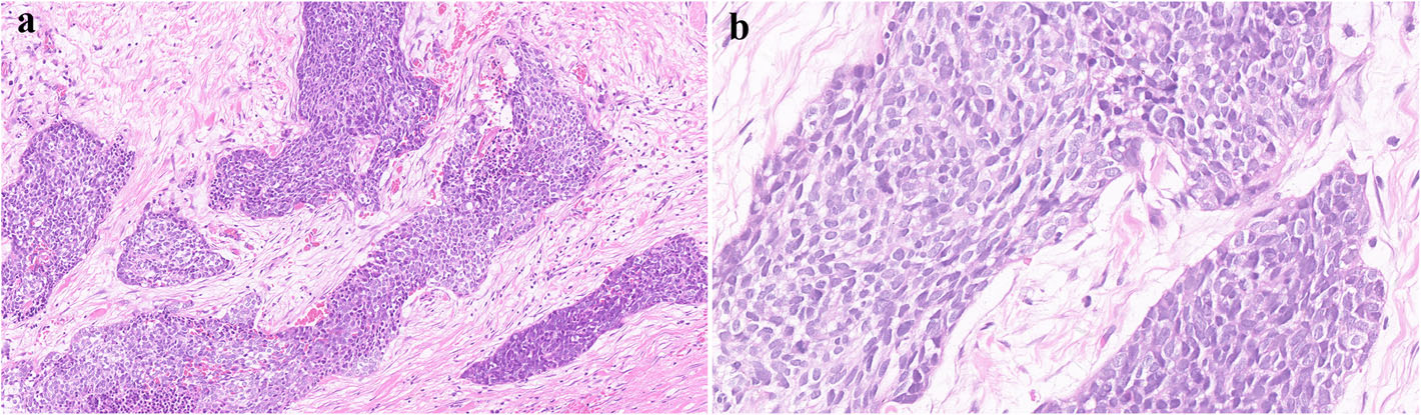
**a** Tumor cells were irregular sheet-like and nest-like distribution, surrounded by proliferative fibrous stroma (H&E, magnification × 200). **b** The tumor cells are small round or oval, with few cytoplasm, unclear cell boundaries, round or oval hyperchromatic nuclei and unclear nucleoli (H&E, magnification × 400)

**Fig. 3:**
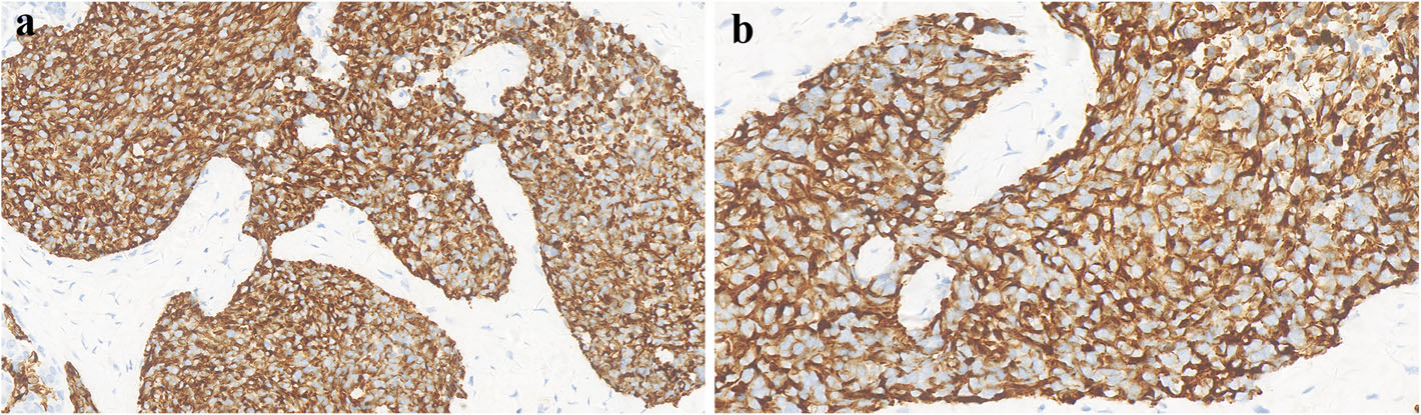
Neoplastic cells showed a diffuse perinuclear staining pattern with desmin (**a** magnification× 400 and **b** magnification × 600)

**Fig. 4:**
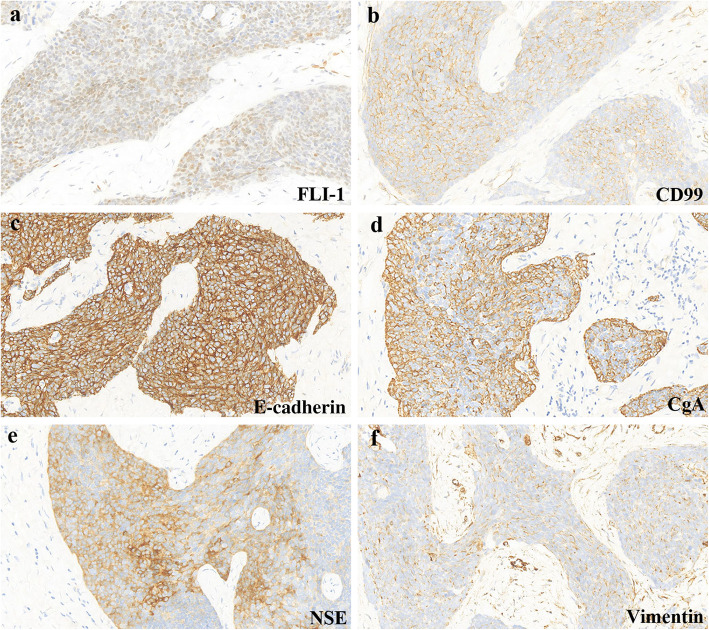
Neoplastic cells are positive for FLI-1 (**a**), CD99 (**b**), E-cadherin (**c**), chromogranin A (CgA) (**d**), neuron specific enolase (NSE) (**e**), and vimentin (**f**)

**Fig. 5:**
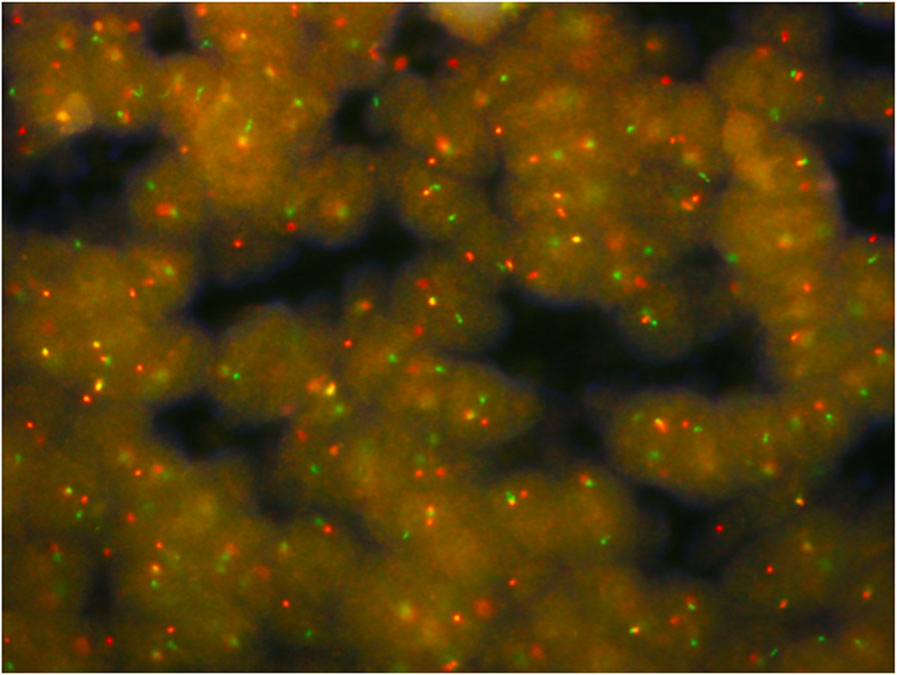
Dual Color Break Apart specific locus FISH probe targeting *EWSR1* gene; green and red signals mark the 5′ and 3′ ends of the gene respectively

Histological examination showed sheets, cords, and nests of small round cells separated focally by desmoplastic stroma (Fig. 2a). Under higher magnification, tumor cells showed round to oval hyperchromatic nuclei with an increased nuclear/cytoplasmic ratio and inconspicuous nucleoli. The cytoplasm of the tumor cells was scanty with indistinct cytoplasmic borders (Fig. 2b). Mitotic activity and individual cell necrosis were common. Immunohistochemical analysis was performed using formalin-fixed paraffin embedded sections from representative tumor blocks and the antibodies listed in Table 1. Immunohistochemical results indicated the multi-directional differentiation of tumor cells. The immunohistochemistry results were as follows: desmin (+) (Fig. 3), FLI-1 (+), CD99 (+), E-cadherinD (+), chromogranin-A (+), neuron-specific enolase (+), vimentin (+) (Fig. 4), pan-cytokeratin (+), epithelial membrane antigen (+), CD56 (+), synaptophysin (weakly positive [+/−]), NKX2.2 (−), WT1 (−), myogenin (−), and S-100 (−). Moreover, the Ki-67 proliferation index was estimated as 50%. The tumor cells were negative for Epstein-Barr virus-encoded small RNA on fluorescence in situ hybridization (FISH). The FISH analysis with a break-apart probe proved that there was *EWSR1* gene spilt in the neoplastic cells (Fig. 5). However, *EWSR1-WT1* fusion detection by reverse transcription-polymerase chain reaction was not performed owing to certain limitations. Based on the above findings, primary lesions in the abdominal cavity and pelvic cavity were excluded, and a final diagnosis of primary DSRCT in the submandibular gland was made.

**Table 1:** List of immunohistochemical antibodies used in diagnosis

Antibody	Clone	Dilution	Source	Result
AR	EP120	Ready-to-use	Maixin China	+
CD56	MX039	Ready-to-use	Maixin China	+
CD99	O13	Ready-to-use	Maixin China	+
CK-Pan	AE1/AE3	Ready-to-use	Maixin China	+
chromogranin A	193A4C7	Ready-to-use	Baidao China	+
desmin	MX046	Ready-to-use	Maixin China	+
EMA	757F5D6	Ready-to-use	Baidao China	+
FLI1	365H5A6	Ready-to-use	Baidao China	+
E-cadherin	MX020	Ready-to-use	Maixin China	+
NSE	5E2	Ready-to-use	Zhongshan China	+
synaptophysin	214A4G5	Ready-to-use	Baidao China	Focal+
vimentin	MX034	Ready-to-use	Maixin China	+
CD117	YR145	Ready-to-use	Maixin China	–
CK20	120B1A5	Ready-to-use	Baidao China	–
CK5/6	MX040	Ready-to-use	Maixin China	–
ERG	MXR004	Ready-to-use	Maixin China	–
HHF35	HHF35	Ready-to-use	Zhongshan China	–
Melan-A	A103	Ready-to-use	Maixin China	–
myogenin	F5D	Ready-to-use	Maixin China	–
NKX2.2	EP336	Ready-to-use	Maixin China	–
P63	MX013	Ready-to-use	Maixin China	–
S-100	503F1E9	Ready-to-use	Baidao China	–
TTF-1	MX011	Ready-to-use	Maixin China	–
WT1	MX012	Ready-to-use	Maixin China	–
Ki-67	MX006	Ready-to-use	Maixin China	50%

*CK-Pan* Pan cytokeratin, *EMA* Epithelial membrane antigen, *NSE* Neuron specific enolase

+: Positive, −: Negative

### Follow-up

Comprehensive anti-tumor therapy mainly based on chemotherapy and radiotherapy was first proposed. However, synchronous chemotherapy was not performed owing to the risk of bone marrow suppression. Therefore, cyclophosphamide combined with doxorubicin and vincristine chemotherapy was used for maintenance treatment. The patient is currently alive and well with no evidence of tumor recurrence.

## Discussion

DSRCT, which was first described as a specific disease by Gerald and Rosai [16, 17], is a rare and aggressive soft-tissue sarcoma. Generally, DSRCT develops on the serosal surface of the abdominal cavity [8–11], but it can also be found in the lung, eye and salivary gland [18–26]. DSRCT has no specific clinical symptoms. Most patients present with initial symptoms of abdominal pain, constipation, ascites, and vomiting [10–14, 27, 28]. These can be accompanied by the manifestations of cachexia, such as fatigue and emaciation. Patients may develop intestinal obstruction, hydronephrosis, and urinary tract irritation owing to tumor compression [29]. In our case, a right submandibular mass without apparent clinical manifestations was detected incidentally. DSRCT is often widely disseminated throughout the peritoneal cavity, and some patients may present with metastasis to the lymph nodes, liver, and occasionally the lungs [8, 12, 30]. Hence, its prognosis is exceedingly poor. The clinical features of previously published DSRCT cases in the last 5 years are summarized in Supplementary Table 2, Additional file 1.

Imaging examinations of DSRCT lack characteristic features. Ultrasound examination usually shows a lobulated soft tissue mass with an uneven internal echo [31]. CT usually reveals single or multiple lobular nodules or lumpy soft tissue masses, with an uneven density of the tumor body and multiple spotted calcifications [10]. The lesions tend to crowd out, surround, and invade the surrounding tissues [19]. DSRCT is usually accompanied by flakes of low intensity when there is a necrotic area in the tumor. Enhanced CT presents mild uneven enhancement and edge enhancement may be observed in some larger masses. Moreover, positron emission tomography (PET)-CT has the potential to monitor residual disease and detect relapse or tumor progression in the early stages [32]. Imaging findings are non-specific, but they can indicate the location, size, and the number of tumors, thereby contributing to biopsy, surgery, and radiotherapy.

The definitive diagnosis of DSRCT is based on typical morphological and immunohistochemical features, especially distinctive molecular characteristics. The pathological and molecular features of previously published cases of DSRCT in the last 5 years are summarized in Supplementary Table 3, Additional file 2. Histologically, the tumor consists of small round cells and peripheral desmoplastic stroma, with occasional cystic degeneration and hemorrhagic necrosis. The tumor has a variegated histology revealing rhabdoid, clear or pleomorphic cells, in addition to typical small round cell morphology. Moreover, the tumor can have intermittent areas of primitive tubules or rosette-like structures [12]. Immunohistochemically, tumor cells show a pattern of multiphenotypic differentiation [8, 15, 33, 34]. This multiple antigen expression profile is a characteristic of DSRCT and can be used to distinguish DSRCT from the other histologically related small round cell tumors. Further, para-nuclear dotted desmin positivity has important diagnostic significance. However, in our case, immunohistochemical staining showed a diffuse perinuclear staining pattern with desmin, while the characteristic dotted positivity was not prominent. Almost all cases of DSRCT are positive for WT1 with cytoplasmic and paranuclear staining pattern. Although the immunohistochemical analysis of classic cases of DSRCT tends to reveal WT1 positivity, N- and C-terminals may be useful as a form of “molecular immunohistochemistry” to identify the EWS–WT1 transcript, as the immunostaining pattern may be altered by variant transcripts and WT1 immunostaining may be negative (as in our case) [35–37]. To establish a DSRCT diagnosis, the interpretation of WT1 immunostaining requires information of antibody target epitopes and correlations with clinical, morphological, and molecular findings. DSRCT is distinguished by the t (11;22) (p13; q12) chromosomal translocation involving a fusion between the transcriptional activating domain of *EWSR1* and the *WT1* gene [38–40]. Studies have also suggested that the *EWSR1-WT1* fusion protein can induce the expression of platelet-derived growth factor-A, which can induce the growth and proliferation of fibroblasts and the production of collagenous stroma; this may explain the characteristic reactive fibrosis of DSRCT [41]. Downstream activation of *EWSR1-WT1* gene fusion includes signaling pathways of vascular endothelial growth factor, IL2RB, and insulin growth factor-1 [42–44]. A better understanding of the effects of these target genes will provide avenues for future treatment.

In the present case, the tumor was composed of nests of small to medium-sized cells, which might be misdiagnosed as small cell carcinoma. Small cell carcinoma can also show the immunoreactivity for epithelial and neuroendocrine markers. However, in our case, the co-expression of epithelial membrane antigen, vimentin and desmin by tumor cells strongly supports a diagnosis of DSRCT. DSRCT should also be distinguished from other carcinomas, such as malignant melanoma, malignant lymphoma, and metastatic neuroblastoma. The current case was negative for Melan-A and S100, which ruled out the likelihood of malignant melanoma. The positivity for epithelial markers helped rule out the possibility of malignant lymphoma, which often involves the lymph nodes, bone marrow and peripheral blood. In addition, stroma of massive nerve fiber network is a characteristic feature of neuroblastoma, which might be a diagnostic clue. Owing to the histological features of small round cells in the present tumor, it must be distinguished from other small round cell tumors, such as rhabdomyosarcoma, primitive neuroectodermal tumor (PNET), and Ewing sarcoma (EWS). Rhabdomyosarcoma is more common in children; the tumor cells are commonly positive for myogenic markers (such as MYOD1 and myogenin), but negative for epithelial and neuroendocrine markers. Morphologically, DSRCT and PNET reveal chrysanthemum-like structure, and both of them are positive for CD99 and neuron-specific enolase. In our case, immunohistochemical results showed positivity for desmin and epithelial markers, which favors a diagnosis of DSRCT over that of PNET. EWS shares histological and immunophenotypic similarities with DSRCT. However, the survival rate of patients with DSRCT is significantly lower than that of EWS patients, which indicates that DSRCT and EWS have different biological backgrounds. EWS mainly occurs in children and is common in bones. Soft tissue involvement is rare. EWS can also be positive for cytokeratin, desmin, CD99, FLI-1, and neuroendocrine markers, which may result in confusion. The diffuse membranous positivity for CD99 is typical of EWS, but in our case, nonspecific cytoplasmic positivity for CD99 is one of the features of DSRCT. Furthermore, negative NKX2.2 is an important clue to distinguish EWS. As both EWS and DSRCT harbor *EWSR1* rearrangements, the break-apart FISH assay for *EWSR1* will not be helpful in the differential diagnosis. However, characteristic translocation of EWS involves *EWSR1* and the ETS family of transcription factors, not *WT1.* Convincingly, documentation of *EWSR1-WT1* fusion is the “gold standard” for the diagnosis of DSRCT [15, 27, 33]. It was not performed in our case because the sample tissue did not meet the requirements and the patient was unwilling; this is a limitation of this case. Taken together, combined with the tumor location and morphological features, as well as a distinctive pattern of multiphenotypic differentiation on immunohistochemistry, the diagnosis of DSRCT was considered. Similar to DSRCT, both myoepithelial carcinoma and synovial sarcoma are also multiphenotypic and expresse multilineage markers. Myoepithelial carcinoma expresses cytokeratin and myogenic markers, such as myogenin, smooth muscle actin and HHF35. The present case showed positivity for desmin and negativity for all other myogenic markers, P63 and CK5/6, strongly favoring a diagnosis of DSRCT. Synovial sarcoma mainly occurs in the extremities. It expresses epithelial and mesenchymal markers, but desmin positivity is uncommon, which can help in the differentiation.

Most reported cases of DSRCT are diagnosed by the histological analysis of formalin-fixed paraffin embedded tissues, and so far, very few cases have been diagnosed by fine needle aspiration cytology (FNAC) [24, 45–49]. FNAC is a cheap, minimally-invasive, and well-tolerated diagnostic technique [50]. Many soft tissue tumors in any location, especially deep-seated tumors, could be sampled by FNAC [51]. Increasing evidence shows that FNAC can provide rapid and valuable diagnosis in identifying soft tissue malignancy. The cytological smear of DSRCT is characterized by stromal fragments and small, round blue cells with hyperchromatic nuclei [24, 46, 47, 49, 51–53]. However, sometimes no fibrosis fragments can be detected in the smear [48, 54]. Occasionally, apoptotic cells and high mitotic activity are observed [24]. Moreover, multiple ancillary techniques, such as immunohistochemical or molecular procedures, can also be performed, which enormously expand the application field of FNAC in the diagnostic approach to soft tissue tumors [24, 50]. However, accurate diagnosis depends on the comprehensive evaluation of histological characteristics, including the growth pattern of neoplastic cells and stromal characteristics; these features may not be well displayed in cytological biopsies because of the cell dispersion, loss of tissue pattern, and paucity of the specimen [52, 55]. Although FNAC can distinguish benign from malignant tumors, it is difficult to provide accurate subtypes. Indeed, some tissue types may lack characteristic morphological or molecular markers, and an accurate diagnosis can only be obtained through surgical resection and histological examination. Therefore, incisional biopsy is still considered the most accurate procedure for diagnosis. In our case, the mass was superficially located and mobile without adhesion. FNAC may not be beneficial in case of such an easily resectable tumor, as complete resection is not only a diagnostic method but also a therapeutic approach. As our patient was reluctant to undergo preoperative FNAC, we routinely performed surgical resection of the mass. We believe that FNAC may emerge as an effective diagnostic tool in the future.

Despite multimodal treatment, DSRCT is highly aggressive and has a poor prognosis. The overall survival in patients is < 3–5 years after diagnosis, and the 5-year survival rate is < 20% [12, 15, 37, 56]. There is no standardized approach for the treatment of this malignant disease. Effective cytoreduction combined with comprehensive therapies, as the best treatment strategy presented in most studies, may improve patient survival rate [14, 15, 28, 33, 34, 57]. With the in-depth analysis of molecular genetics of DSRCT, targeted therapy, immunotherapy, and other methods have been gradually applied to the treatment of DSRCT in recent years [58, 59].

## Conclusions

In summary, DSRCT is a poorly understood malignant tumor with characteristic morphology, immunophenotype, and cytological features. The disease does not present with specific clinical signs or symptoms. PET-CT may help diagnose recurrent disease at an early stage. The *EWSR1-WT1* fusion detection by reverse transcription-polymerase chain reaction is the gold standard for the diagnosis of DSRCT. When it is not feasible, as in our case, definitive diagnosis mainly depends on a comprehensive analysis of histological and immunohistochemical studies. The submandibular gland is an unusual site for DSRCT, suggesting that the tumor may not have a site-specific predilection. Therefore, pathologists and clinicians have to be aware of its possible occurrence in extra-peritoneal regions. To better diagnose this rare and intriguing disease, further studies are needed in future.

## Supplementary Information

**Additional file 1.**

**Additional file 2.**

## Data Availability

All data generated or analyzed during this case are included within the article.

## Abbreviations

DSRCT: Desmoplastic small round cell tumor
CT: Computed tomography
PET: Positron emission tomography
FISH: Fluorescence in situ hybridization
PNET: Primitive neuroectodermal tumor
EWS: Ewing sarcoma
FNAC: Fine needle aspiration cytology
