# Supplementary material for: Primary desmoplastic small round cell tumor of the submandibular gland: a case report and literature review
Source: Diagn Pathol. 2022 Jan 7;17:6. doi: 10.1186/s13000-021-01183-3 (PMC8742402; doi:10.1186/s13000-021-01183-3)
Supplement: Supplementary file 1 — Additional file 1. [file 13000_2021_1183_MOESM1_ESM.doc]

| **Table 2. Clinical features of previously reported and present cases of DSRCT in recent 5 years** | | | | | | | |
| --- | --- | --- | --- | --- | --- | --- | --- |
| Sites | Age (y)/Sex | Clinical symptoms | Maximum diameter of tumor (cm) | Sites of metastases | Treatment | Follow-up (mo) | Ref |
| Abdomen and pelvis | 35/M | Flatulence, abdominal pain.et al | >10 | NR | Sg, CT | DOD-5 | 1 |
|  | 31/M | Paroxysmal epigastric pain.et al | NR | Liver, bone, and lung, multiple LNs | Sg, CT | AWD-8 | 2 |
|  | 38/M | Anterior abdominal wall nodule | >5 | Right inguinal and omental LN | Sg, CT | AWD-4 | 3 |
|  | 25/M | Constipation, bloating.et al | NR | NR | Sg | NR | 4 |
|  | 20/M | Abdominal discomfort.et al | NR | multiple  pelvic intraperitoneal metastases | Sg, CT | AWD | 5 |
| Ovarian | 19/F | Secondary amenorrhea | >15 | NR | Sg | DOD-3 | 6 |
|  | 19/F | Abdominal fullness | >15 | Sigmoid colon, omentum and cul-de-sac | Sg, CT | AWD-40 | 7 |
|  | 31/F | Abdominal discomfort.et al | >10 | Bowel, liver and stomach, and subdiaphragmatic peritoneum | Sg | DOD-1 | 8 |
|  | 22/F | Abdominal pain | NR | NR | Sg, BT and CT | AWD | 9 |
|  | 23/F | Abdominal pain, nausea.et al | >5 | Liver, peritoneal wall, omentum, uterus, and bowel | Sg, CT | DOD-4 | 10 |
| Kidney | 8/F | Abdominal pain | >10 | Lung, liver, adrenal and LN | Sg, CT | DOD-30 | 11 |
|  | 15/M | Hematuria | >10 | Adrenal, liver, lung, and bone | Sg, CT and RT | DOD-48 | 12 |
| Thorax | 51/M | Shortness of breath, chest tightness.et al | NR | Brain parenchyma and left adrenal | Sg, CT and RT | DOD-17 | 13 |
|  | 3/M | Cough, fever, fatigue.et al | NR | NR | Sg, CT | NR | 14 |
| Pancreas | 9/M | Abdominal pain | >15 | Mesenteric LNs | Sg, CT | AWD | 15 |
| Superficial location | 15/M | Left shoulder and lower back pain | <5 | NR | Sg, CT | AFD-48 | 16 |
| Bowel | 30/F | Abdominal discomfort and fatigue | >10 | NR | Sg | AFD-6 | 17 |
| Eye | 16/M | Pain swelling and a nodule in left orbit | <5 | NR | Sg | AFD-24 | 18 |
|  | 2m/M | Swelling inferior to his right orbit | <5 | NR | Sg, CT | AWD-12 | 19 |
|  | 16/M | A left eye mass | NR | NR | Sg | AFD-12 | 20 |
|  | 4/M | Swelling of the right eye | <5 | NR | Sg, CT | AFD-12 | 21 |
| Sinus | 55/F | Right eye epiphora and nasal bleeding | NR | Bone marrow | Sg, CT and RT | DOD-2 | 22 |
| Bone | 33/M | Intermittent pain in the left lower limb | >5 | NR | Sg, CT | AFD-18 | 23 |
| Brachial plexus | 42/F | A mass in left armpit | >5 | Lung, LNs | Sg, CT and RT | AWD-56 | 24 |
| Ear | 59/M | Suppuration of right ear.et al | NR | NR | Sg, CT and RT | AFD-48 | 25 |
| Testis | 14/M | A left groin bulge | NR | NR | Sg, CT and RT | AFD-24 | 26 |
| Lymph node | 11/M | A left neck-mass | <5 | Cervical LNs | Sg, BT and CT | AFD-39 | 27 |
| Cranium | 27/M | Lethargy, loss of libido.et al | NR | Mediastinal, subcarinal, and hilar LNs | Sg | DOD-20 | 28 |
| Salivary gland | 38/M | A right neck mass | >5 | Ipsilateral neck LN | Sg, CT and RT | AWD-5 | 29 |
|  | 18/M | A mass in the left parotid gland | <5 | Retropharyngeal LN | Sg, CT and RT | NR | 30 |
|  | 17/M | A lesion in left parotid gland | <5 | LN | Sg, CT and RT | AWD-17 | 31 |
|  | 49/M | A mass in the right parotid gland | <5 | NR | Sg, RT | AFD-36 | 32 |
|  | 10/M | A small mass in submandibular region | NR | Ipsilateral cervical LNs | Sg, CT and RT | AFD | 33 |
| Present case | 26/M | A mass in the right submandibular gland | <5 | NR | Sg, CT and RT | AFD-24 |  |
| M: male, F: female, NR: not reported, DOD: died of disease, AWD: alive with disease, AFD: alive free of disease  LNs, lymph node; Sg, Surgery; CT, chemotherapy; RT, radiation therapy; BT, beam therapy | | | | | | | |
|  |  |  |  |  |  |  |  |

1. Tsoukalas, N.; Kiakou, M.; Nakos, G.; Tolia, M.; Galanopoulos, M.; Tsapakidis, K.; Kamposioras, K.; Christofyllakis, C.; Dimitrakopoulos, G.; Sambaziotis, D., Desmoplastic small round-cell tumour of the peritoneal cavity: case report and literature review. *Ann R Coll Surg Engl* **2020,** *102* (4), e77-e81.

2. Tian, Y.; Cheng, X.; Li, Y., Chemotherapy combined with apatinib for the treatment of desmoplastic small round cell tumors: A case report. *J Cancer Res Ther* **2020,** *16* (5), 1177-1181.

3. Chen, H. M.; Feng, G., Use of anlotinib in intra-abdominal desmoplastic small round cell tumors: a case report and literature review. *Onco Targets Ther* **2019,** *12*, 57-61.

4. Ofori, E.; Ramai, D.; Nigar, S.; Xiao, P.; Shahzad, G.; Reddy, M., Desmoplastic Small Round Cell Tumor: A Rare Case of Extraluminal Bowel Obstruction and Review of the Literature. *J Gastrointest Cancer* **2019,** *50* (3), 560-563.

5. Butt, S. U.; Bull, J. M. H.; Scott, A., Desmoplastic Small Round-Cell Tumor in a Young Indigenous Australian Man: A Case Report. *J Glob Oncol* **2017,** *3* (1), 79-81.

6. Phulware, R. H.; Roy, M.; Singh, N.; Kumar, S.; Mathur, S. R., Desmoplastic small round cell tumor of the ovary: A rare but poor prognostic disease in a young woman! *Indian J Pathol Microbiol* **2021,** *64* (1), 206-209.

7. Vujić, G.; Mikuš, M.; Matak, L.; Bonevski, A.; Babić, I.; Planinić, P.; Babić, D.; Ćorušić, A., Desmoplastic Small Round Cell Tumor of the Ovary: A Case Report with a New Modality of Treatment and Review of the Literature. *Rev Bras Ginecol Obstet* **2020,** *42* (5), 297-302.

8. Lee, S. Y.; Koo, Y. J.; Lee, D. H., Desmoplastic small round cell tumour with ovarian involvement: a case report. *J Obstet Gynaecol* **2020,** *40* (1), 141-142.

9. Atef, A.; Gaballa, K.; Zuhdy, M.; Atallah, K.; Elkashef, W.; Awny, S.; Gadelhak, B.; Refky, B., Primary desmoplastic small-round-cell tumor of the ovary. *J Egypt Natl Canc Inst* **2019,** *31* (1), 4.

10. Altal, O. F.; Aleshawi, A. J.; Tashtush, N. A.; Alhowary, A., A 23-Year-Old Joradanian Woman with a Desmoplastic Small Round Cell Tumor Involving the Ovary. *Am J Case Rep* **2019,** *20*, 1675-1678.

11. Ertoy Baydar, D.; Armutlu, A.; Aydin, O.; Dagdemir, A.; Yakupoglu, Y. K., Desmoplastic small round cell tumor of the kidney: a case report. *Diagn Pathol* **2020,** *15* (1), 95.

12. Galliani, C. A.; Bisceglia, M.; Del Giudice, A.; Cretì, G., Desmoplastic Small Round Cell Tumor of the Kidney: Report of a Case, Literature Review, and Comprehensive Discussion of the Distinctive Morphologic, Immunohistochemical, and Molecular Features in the Differential Diagnosis of Small Round Cell Tumors Affecting the Kidney. *Adv Anat Pathol* **2020,** *27* (6), 408-421.

13. Jin, D.; Chen, M.; Wang, B.; Gou, Y., Mediastinal desmoplastic small round cell tumor. *Medicine (Baltimore)* **2020,** *99* (44), e22921.

14. Suhag, S.; Byrd, R. H.; Jaiswal, K., Rare Case of Thoracic Desmoplastic Small Round Cell Tumor in a Three-Year-Old Boy. *J Oncol Pract* **2019,** *15* (11), 617-620.

15. Saleh, D.; Al-Maghrabi, S.; Al-Maghrabi, H.; Al-Maghrabi, J., Desmoplastic Small Round Cell Tumor of Pancreatic Origin in a Young Child: A Case Report and Review of Literature. *Am J Case Rep* **2020,** *21*, e922762.

16. Asadbeigi, S. N.; Zhang, L.; Linos, K., Subcutaneous desmoplastic small round-cell tumor: An unusual primary location expanding the differential of superficial round-cell tumors. *J Cutan Pathol* **2020,** *47* (8), 768-775.

17. Huang, J.; Sha, L.; Zhang, H.; Tang, X.; Zhang, X., Desmoplastic small round cell tumor in transverse colon: report of a rare case. *Int Surg* **2015,** *100* (5), 809-13.

18. He, X. R.; Liu, Z.; Wei, J.; Li, W. J.; Liu, T., Primary desmoplastic small round cell tumor in the left orbit: a case report and literature review. *Int Ophthalmol* **2019,** *39* (2), 471-475.

19. Huang, A.; Patel, N., Orbital desmoplastic small round cell tumor in an infant. *Digit J Ophthalmol* **2018,** *24* (4), 31-35.

20. Wang, P.; Liu, Y.; Liu, X.; Yan, Q.; Wang, L., Solid-pattern desmoplastic small round cell tumor of the orbit: a case report. *Int J Clin Exp Pathol* **2018,** *11* (5), 2864-2868.

21. Bengu Cobanoglu, H.; Hanna, E. Y.; Bell, D.; Esmaeli, B., Desmoplastic Small Round Cell Tumor Presenting as an Ocular Mass: Unusual Localization and Remarkable Surgical Approach. *Curr Oncol Rep* **2017,** *19* (12), 80.

22. Tao, Y.; Shi, L.; Ge, L.; Yuan, T.; Shi, L., Sinonasal desmoplastic small round cell tumor: a case report and review of the literature. *BMC Cancer* **2019,** *19* (1), 868.

23. Xuesong, D.; Hong, G.; Weiguo, Z., Primary desmoplastic small round cell tumor of the tibia: PET/CT and MRI presentation of a rare case and review of the literature. *J Bone Oncol* **2020,** *20*, 100272.

24. Guedes-Corrêa, J. F.; Amorim, R. P.; Pereira, M.; Cardoso, R. S. V.; Costa, F. D.; Bianchi, B. S.; Siquara-de-Souza, A. C., Multimodal treatment of an extremely rare desmoplastic small round cell tumor primary to the brachial plexus - A case report and review of literature. *Surg Neurol Int* **2019,** *10*, 140.

25. Xu, J.; Yao, M.; Yang, X.; Liu, T.; Wang, S.; Ma, D.; Li, X., Desmoplastic small round cell tumor of the middle ear: A case report. *Medicine (Baltimore)* **2018,** *97* (17), e0494.

26. Sedig, L.; Geiger, J.; Mody, R.; Jasty-Rao, R., Paratesticular desmoplastic small round cell tumors: A case report and review of the literature. *Pediatr Blood Cancer* **2017,** *64* (12).

27. Faras, F.; Abo-Alhassan, F.; Hussain, A. H.; Sebire, N. J.; Al-Terki, A. E., Primary desmoplastic small round cell tumor of upper cervical lymph nodes. *Oral Surg Oral Med Oral Pathol Oral Radiol* **2015,** *120* (1), e4-e10.

28. Thondam, S. K.; du Plessis, D.; Cuthbertson, D. J.; Das, K. S.; Javadpour, M.; MacFarlane, I. A.; Leggate, J.; Haylock, B.; Daousi, C., Intracranial desmoplastic small round cell tumor presenting as a suprasellar mass. *J Neurosurg* **2015,** *122* (4), 773-7.

29. Cai, Z.; Zhang, L.; Karni, R. J.; Saluja, K.; Liu, J.; Zhu, H., Desmoplastic Small Round Cell Tumor of Parotid Gland: A Rare Entity With Diagnostic Challenge. *Int J Surg Pathol* **2020,** *28* (7), 782-786.

30. Ninchritz-Becerra, E.; González-García, J.; García-Iza, L.; Chiesa Estomba, C. M., Sr., Desmoplastic Small Round Cell Tumor: A Rare Location in the Parotid Gland. *Cureus* **2020,** *12* (8), e10068.

31. Lozano, M. D.; Landa, A.; Tobar, L. G.; De Andrea, C.; Larrache, J.; Echeveste, J. I.; Paricio, J. J.; Sánchez, B.; Medina, A.; Paisan, A., A comprehensive diagnosis of a desmoplastic small round cell tumor of unusual location based on fine-needle aspiration cytology: Report of a case arising in the parotid gland and review of the literature. *Diagn Cytopathol* **2020,** *48* (9), 827-832.

32. Hatanaka, K. C.; Takakuwa, E.; Hatanaka, Y.; Suzuki, A.; S, I. I.; Tsushima, N.; Mitsuhashi, T.; Sugita, S.; Homma, A.; Morinaga, S.; Hashegawa, T.; Matsuno, Y., Desmoplastic small round cell tumor of the parotid gland-report of a rare case and a review of the literature. *Diagn Pathol* **2019,** *14* (1), 43.

33. Rubinstein, J. D.; Gupta, A.; Szabo, S.; Pressey, J. G., A case of submandibular desmoplastic small round cell tumor: Diagnostic and management approaches to an atypical presentation of a rare tumor. *Pediatr Blood Cancer* **2020,** *67* (4), e28178.
