# Supplementary material for: Primary desmoplastic small round cell tumor of the submandibular gland: a case report and literature review
Source: Diagn Pathol. 2022 Jan 7;17:6. doi: 10.1186/s13000-021-01183-3 (PMC8742402; doi:10.1186/s13000-021-01183-3)
Supplement: Supplementary file 2 — Additional file 2. [file 13000_2021_1183_MOESM2_ESM.doc]

| **Table 3. Pathological features of previously reported and present cases of DSRCT in recent 5 years** | | | | | | | | | | |
| --- | --- | --- | --- | --- | --- | --- | --- | --- | --- | --- |
| Sites | Age (y)/Sex | Immunohistochemical features | | | | |  | Molecular pathology | Follow-up (mo) |  |
|  |  | Epithelial (EMA/CK) | Mesenchymal  (VIM) | Neural  (CD56/Syn/NSE) | Myogenic (DES/MYO) | CD99 | WT1 |  |  | Ref |
| Abdomen and pelvis | 31/M | +/+ | + | NR/–/+ | +/NR | NR | – | DNA llumina high-throughput sequencing | AWD-8 | 1 |
|  | 20/M | +/+ | + | NR/NR/NR | +/NR | NR | – | FISH | AWD | 2 |
| ovarian | 19/F | +/NR | + | NR/NR/– | +/NR | NR | + | RT-PCR | AWD-40 | 3 |
| kidney | 8/F | +/+ | + | +/–/– | +/NR | – | – | FISH | DOD-30 | 4 |
|  | 15/M | +/+ | + | NR/+/+ | +/NR | + | + | FISH and RT-PCR | DOD-48 | 5 |
| Thorax | 3/M | +/+ | + | +/–/+ | +/– | + | – | Evaluation of EWSWT1 translocation | NR | 6 |
| Pancreas | 9/M | +/– | NR | +/–/+ | +/– | + | NR | RT-PCR | AWD | 7 |
| Superficial location | 15/M | +/NR | + | NR/–/NR | +/NR | NR | – | FISH | AFD-48 | 8 |
| Eye | 16/M | NR/NR | + | +/+/+ | +/NR | + | NR | FISH | AFD-24 | 9 |
|  | 2m/M | NR/– | NR | NR/–/NR | +/NR | + | + | Comprehensive genomic profiling | AWD-12 | 10 |
|  | 16/M | –/– | NR | NR/+/+ | +/– | + | – | FISH | AFD-12 | 11 |
| Bone | 33/M | NR/+ | NR | +/–/NR | +/– | + | + | Molecular analysis | AFD-18 | 12 |
| Brachial plexus | 42/F | NR/+ | NR | +/–/NR | +/– | + | + | FISH | AWD-56 | 13 |
| Testis | 14/M | +/+ | NR | NR/NR/+ | +/+ | + | NR | RT-PCR | AFD-24 | 14 |
| Lymph node | 11/M | +/+ | + | –/NR/+ | +/– | – | – | FISH and RT-PCR | AFD-39 | 15 |
| Cranium | 27/M | +/+ | + | –/–/+ | +/NR | – | – | FISH and RT-PCR | DOD-20 | 16 |
| Salivary gland | 38/M | NR/NR | NR | NR/–/NR | +/NR | – | NR | FISH | AWD-5 | 17 |
|  | 18/M | NR/+ | NR | NR/NR/NR | +/– | – | NR | FISH | NR | 18 |
|  | 17/M | NR/+ | NR | NR/NR/NR | +/– | – | NR | FISH | AWD-17 | 19 |
|  | 49/M | +/+ | + | +/–/NR | +/NR | – | + | FISH and RT-PCR | AFD-36 | 20 |
|  | 10/M | NR/NR | NR | NR/NR/NR | NR/NR | NR | + | Next-generation sequencing of DNA | AFD | 21 |
| Present case | 26/M | +/+ | + | +/+/+ | +/– | + | – | FISH | AFD-24 |  |
| EMA: Epithelial membrane antigen, CK: Cytokeratin, VIM: Vimentin, SYN: synaptophysin, NSE: Neuron specific enolase, DES: Desmin, MYO: myogenin  M: male, F: female, NR: not reported, DOD: died of disease, AWD: alive with disease, AFD: alive free of disease  FISH: Fluorescence in situ hybridization, RT-PCR: Reverse transcription-polymerase chain reaction | | | | | | | | | | |
|  |  |  |  |  |  |  |  |  |  |  |

1. Tian, Y.; Cheng, X.; Li, Y., Chemotherapy combined with apatinib for the treatment of desmoplastic small round cell tumors: A case report. *J Cancer Res Ther* **2020,** *16* (5), 1177-1181.

2. Butt, S. U.; Bull, J. M. H.; Scott, A., Desmoplastic Small Round-Cell Tumor in a Young Indigenous Australian Man: A Case Report. *J Glob Oncol* **2017,** *3* (1), 79-81.

3. Vujić, G.; Mikuš, M.; Matak, L.; Bonevski, A.; Babić, I.; Planinić, P.; Babić, D.; Ćorušić, A., Desmoplastic Small Round Cell Tumor of the Ovary: A Case Report with a New Modality of Treatment and Review of the Literature. *Rev Bras Ginecol Obstet* **2020,** *42* (5), 297-302.

4. Ertoy Baydar, D.; Armutlu, A.; Aydin, O.; Dagdemir, A.; Yakupoglu, Y. K., Desmoplastic small round cell tumor of the kidney: a case report. *Diagn Pathol* **2020,** *15* (1), 95.

5. Galliani, C. A.; Bisceglia, M.; Del Giudice, A.; Cretì, G., Desmoplastic Small Round Cell Tumor of the Kidney: Report of a Case, Literature Review, and Comprehensive Discussion of the Distinctive Morphologic, Immunohistochemical, and Molecular Features in the Differential Diagnosis of Small Round Cell Tumors Affecting the Kidney. *Adv Anat Pathol* **2020,** *27* (6), 408-421.

6. Suhag, S.; Byrd, R. H.; Jaiswal, K., Rare Case of Thoracic Desmoplastic Small Round Cell Tumor in a Three-Year-Old Boy. *J Oncol Pract* **2019,** *15* (11), 617-620.

7. Saleh, D.; Al-Maghrabi, S.; Al-Maghrabi, H.; Al-Maghrabi, J., Desmoplastic Small Round Cell Tumor of Pancreatic Origin in a Young Child: A Case Report and Review of Literature. *Am J Case Rep* **2020,** *21*, e922762.

8. Asadbeigi, S. N.; Zhang, L.; Linos, K., Subcutaneous desmoplastic small round-cell tumor: An unusual primary location expanding the differential of superficial round-cell tumors. *J Cutan Pathol* **2020,** *47* (8), 768-775.

9. He, X. R.; Liu, Z.; Wei, J.; Li, W. J.; Liu, T., Primary desmoplastic small round cell tumor in the left orbit: a case report and literature review. *Int Ophthalmol* **2019,** *39* (2), 471-475.

10. Huang, A.; Patel, N., Orbital desmoplastic small round cell tumor in an infant. *Digit J Ophthalmol* **2018,** *24* (4), 31-35.

11. Wang, P.; Liu, Y.; Liu, X.; Yan, Q.; Wang, L., Solid-pattern desmoplastic small round cell tumor of the orbit: a case report. *Int J Clin Exp Pathol* **2018,** *11* (5), 2864-2868.

12. Xuesong, D.; Hong, G.; Weiguo, Z., Primary desmoplastic small round cell tumor of the tibia: PET/CT and MRI presentation of a rare case and review of the literature. *J Bone Oncol* **2020,** *20*, 100272.

13. Guedes-Corrêa, J. F.; Amorim, R. P.; Pereira, M.; Cardoso, R. S. V.; Costa, F. D.; Bianchi, B. S.; Siquara-de-Souza, A. C., Multimodal treatment of an extremely rare desmoplastic small round cell tumor primary to the brachial plexus - A case report and review of literature. *Surg Neurol Int* **2019,** *10*, 140.

14. Sedig, L.; Geiger, J.; Mody, R.; Jasty-Rao, R., Paratesticular desmoplastic small round cell tumors: A case report and review of the literature. *Pediatr Blood Cancer* **2017,** *64* (12).

15. Faras, F.; Abo-Alhassan, F.; Hussain, A. H.; Sebire, N. J.; Al-Terki, A. E., Primary desmoplastic small round cell tumor of upper cervical lymph nodes. *Oral Surg Oral Med Oral Pathol Oral Radiol* **2015,** *120* (1), e4-e10.

16. Thondam, S. K.; du Plessis, D.; Cuthbertson, D. J.; Das, K. S.; Javadpour, M.; MacFarlane, I. A.; Leggate, J.; Haylock, B.; Daousi, C., Intracranial desmoplastic small round cell tumor presenting as a suprasellar mass. *J Neurosurg* **2015,** *122* (4), 773-7.

17. Cai, Z.; Zhang, L.; Karni, R. J.; Saluja, K.; Liu, J.; Zhu, H., Desmoplastic Small Round Cell Tumor of Parotid Gland: A Rare Entity With Diagnostic Challenge. *Int J Surg Pathol* **2020,** *28* (7), 782-786.

18. Ninchritz-Becerra, E.; González-García, J.; García-Iza, L.; Chiesa Estomba, C. M., Sr., Desmoplastic Small Round Cell Tumor: A Rare Location in the Parotid Gland. *Cureus* **2020,** *12* (8), e10068.

19. Lozano, M. D.; Landa, A.; Tobar, L. G.; De Andrea, C.; Larrache, J.; Echeveste, J. I.; Paricio, J. J.; Sánchez, B.; Medina, A.; Paisan, A., A comprehensive diagnosis of a desmoplastic small round cell tumor of unusual location based on fine-needle aspiration cytology: Report of a case arising in the parotid gland and review of the literature. *Diagn Cytopathol* **2020,** *48* (9), 827-832.

20. Hatanaka, K. C.; Takakuwa, E.; Hatanaka, Y.; Suzuki, A.; S, I. I.; Tsushima, N.; Mitsuhashi, T.; Sugita, S.; Homma, A.; Morinaga, S.; Hashegawa, T.; Matsuno, Y., Desmoplastic small round cell tumor of the parotid gland-report of a rare case and a review of the literature. *Diagn Pathol* **2019,** *14* (1), 43.

21. Rubinstein, J. D.; Gupta, A.; Szabo, S.; Pressey, J. G., A case of submandibular desmoplastic small round cell tumor: Diagnostic and management approaches to an atypical presentation of a rare tumor. *Pediatr Blood Cancer* **2020,** *67* (4), e28178.
